# P-1255. Dosing of Piperacillin in Altered Renal Function with and without Renal Replacement Therapy for Required Increased Exposition

**DOI:** 10.1093/ofid/ofae631.1437

**Published:** 2025-01-29

**Authors:** Güzin Surat, Emma Dohmann, Oliver Scherf-Clavel, Max Kurlbaum, Stefan Hagel

**Affiliations:** University Hospital Würzburg, Würzburg, Bayern, Germany; Julius-Maximilians-Universität Würzburg, Würzburg, Bayern, Germany; Faculty for Chemistry and Pharmacy, Ludwid-Maximilians-University Munich, Munich, Bayern, Germany; University Hospital Würzburg, Würzburg, Bayern, Germany; University Hospital Jena, Jena, Thuringen, Germany

## Abstract

**Background:**

Piperacillin/ Tazobactam [TZP] is a commonly used antibiotic in critically ill and non-critically ill patients, but not lot is known about TZP pharmacokinetics in non-critically ill patients needing dose reduction due to renal impairment. While Summary of Product Characteristics [SoPC] suggests 16 g Piperacillin daily for severe infections, patients with renal impairment (CrCl < 40 ml/min) are advised by the SoPC to only 8-12 g Piperacillin per day. The aim of this study was to determine the probability of target attainment (PTA), with the PK/PD target being set to unbound concentrations ≥60% of the time above 16 mg/L (MIC of increased exposure pathogen) in patients with decreased renal function especially in consideration of changed definitions on the susceptibility testing by EUCAST advising higher treatment dosing regimens for infections with *Pseudomonas aeruginosa*.

**Figure:**
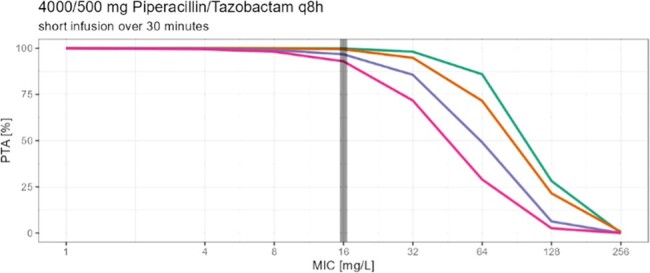

**Methods:**

In this prospective two center study we measured 135 piperacillin serum concentration from 49 patients with decreased renal function to determine peak, trough and concentrations between peak and trough. Specific designed software (Monolix 2023R1) was used to develop a population pharmacokinetic model. The PTA was calculated for four groups (eGFR 40, 30 and 20 ml/min and 10 ml/min with intermitted hemodialysis) with different dosing strategies using Monte Carlo simulation (1000 patients per eGFR group). Covariates like age, sex, height, weight, eGFR, body mass index and body surface area (BSA) were evaluated.

**Figure:**
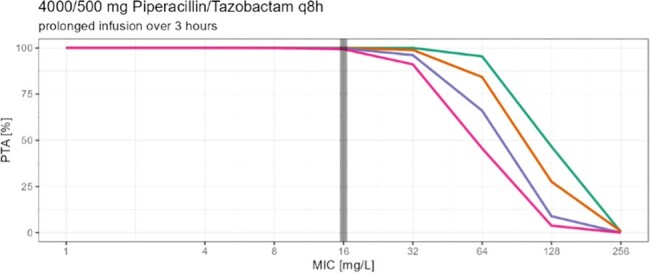

**Results:**

Patients with eGFR 40 and 30 ml/min reached a PTA of over 90% when following the SoPC (eGFR 20-40 ml/min: q8h, 30 min infusions) and increased their PTA to almost 100% when switching to prolonged (over 3h infusions). Patients with eGFR 20 ml/min reached a PTA of almost 90% with a q12h/ 30 min infusion dosage regimen recommended by SoPC for patients with eGFR < 20 ml/min and increased their PTA by about 5% when receiving prolonged infusions (3h). Patients with eGFR of 10 ml/min and intermitted hemodialysis reached a PTA of about 93% (q12h, 30 min infusions) and would reach a PTA of almost 100 % when switching to 3h infusions.

**Figure:**
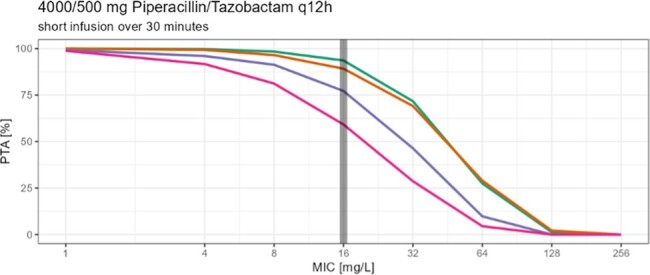

**Conclusion:**

Prolonged infusions increased the PTA in all groups. The results indicate adjustments for Piperacillin infusions in renal impairment.

**Figure:**
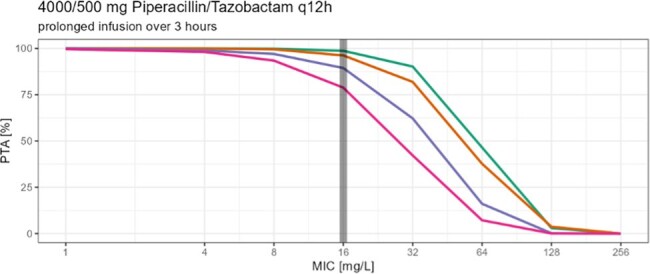

**Disclosures:**

**All Authors**: No reported disclosures

